# Ursodeoxycholic acid relieves clinical severity of COVID-19 in patients with chronic liver diseases

**DOI:** 10.3389/fmed.2025.1494248

**Published:** 2025-02-06

**Authors:** Tiantian Hu, Jie Tong, Yunhui Yang, Changrong Yuan, Jiming Zhang, Jinyu Wang

**Affiliations:** ^1^Department of Infectious Diseases, Shanghai Key Laboratory of Infectious Diseases and Biosafety Emergency Response, National Medical Center for Infectious Diseases, Huashan Hospital, Fudan University, Shanghai, China; ^2^Fudan University School of Nursing, Fudan University, Shanghai, China; ^3^Shanghai Institute of Infectious Diseases and Biosecurity, Key Laboratory of Medical Molecular Virology (MOE/MOH), Shanghai Medical College, Fudan University, Shanghai, China; ^4^Department of Infectious Diseases, Jing’An Branch of Huashan Hospital, Fudan University, Shanghai, China

**Keywords:** chronic liver diseases, SARS-CoV-2, ursodeoxycholic acid, prognosis, clinical outcomes

## Abstract

**Background:**

The potential effect of ursodeoxycholic acid (UDCA) on the clinical outcomes of SARS-CoV-2 in patients with chronic liver diseases has been a subject of ongoing debate since the onset of the SARS-CoV-2 pandemic in 2019. This study aims to investigate the effect of UDCA on the prognosis of SARS-CoV-2 infection in patients with chronic liver diseases.

**Methods:**

A total of 926 patients with chronic liver diseases who contracted their first SARS-CoV-2 infection during December 2022 to January 2023, were included in this study. Participants were divided into two groups based on the use of UDCA: the UDCA cohort (*n* = 329) and the non-UDCA cohort (*n* = 597). After performing a 1:1 age-and sex-matching, the analysis proceeded with 309 patients from each group for further evaluation.

**Results:**

In the UDCA-treated cohort, the incidence of asymptomatic SARS-CoV-2 infections was significantly higher, with 30.1% of patients affected, compared to 6.47% in the non-UDCA group (*p* < 0.0001). Multivariable analysis identified UDCA as a protective factor against symptomatic infections, yielding an odds ratio (OR) of 4.77 (95% CI: 2.70–8.44, *p* < 0.001). Furthermore, age over 50 was found to be a risk factor for asymptomatic infections in the UDCA cohort, with an adjusted OR of 1.51 (95% CI: 1.01–2.24, *p* = 0.05).

**Conclusion:**

The study suggests that UDCA therapy may improve clinical outcomes in patients with chronic liver diseases patients who are infected with SARS-CoV-2, highlighting its potential role in improving prognosis within this vulnerable population. However, further research is required to validate these findings and to elucidate the mechanisms underlying UDCA’s protective effect.

## Introduction

As a public health emergency of international concern caused by the SARS-CoV-2 virus (1), COVID-19 has resulted in over 770 million confirmed cases and more than 6.9 million deaths as of 27 August 2023 (2). Since China relaxed its epidemic prevention measures on December 7, 2022, the infection rate of COVID-19 among Chinese residents has continued to rise (3–5). Notably, patients with chronic liver diseases are increasingly vulnerable to co-infection and exhibit higher mortality (6, 7). Consequently, research on the infection status and preventive measures for patients with chronic liver disease and COVID-19 has become of critical importance (7, 8).

Ursodeoxycholic acid (UDCA) is primarily used in patients with chronic liver diseases, effectively managing hepatitis-related liver dysfunction and cholestatic liver diseases (9). Previous studies have explored the potential benefits of UDCA in a range of liver diseases (10–13), including fatty liver disease, primary biliary cholangitis (PBC), and primary sclerosing cholangitis (PSC). Known for its hepatoprotective properties, UDCA has been shown to improve liver function, reduce inflammation, and enhance bile flow in patients with these diseases (14, 15). Beyond its liver-protective effects, emerging studies have suggested a potential antiviral mechanism by which UDCA exerts its action (16, 17). UDCA had been found to interfere with viral replication and inhibit the entry of viruses into host cells (17, 18). It has demonstrated antiviral activity against several viruses, including hepatitis B (HBV), hepatitis C (HCV), and human immunodeficiency virus (HIV) (16–18). UDCA is believed to modulate immune responses, including increased interferon production and direct inhibition of viral enzymes. However, UDCA is not a conventional antiviral agent, and further research is required to fully elucidate its mechanisms and assess its potential as a therapeutic option for viral infections.

Previous studies have indicated that UDCA is associated with a reduced risk of SARS-CoV-2 infection and less severe COVID-19 outcomes in patients with cirrhosis (19, 20). UDCA has been shown to decrease susceptibility to SARS-CoV-2 by down-regulating angiotensin-converting enzyme 2 (ACE2) (19) and has been identified as a protective factor (20). However, other studies have reported that UDCA did not improve COVID-19 outcomes in hospitalized patients (21, 22). The potential protective role of UDCA remains controversial and warrants further investigation.

In this study, we examined the effects of UDCA treatment on the clinical outcomes of COVID-19 in patients with chronic liver disease. By utilizing an age-and sex-matched cohort, the research aims to inform strategies for managing COVID-19 in this vulnerable population. The findings offer valuable insights into the potential antiviral effects of UDCA.

## Methods

### Study design

Patients with chronic liver diseases, including 458 patients with hepatitis B, 306 with autoimmune hepatitis, 38 with primary biliary cholangitis, 84 with fatty liver and 40 with liver conditions, who were infected with SARS-CoV-2 for the first time during the second Omicron wave in Shanghai, were enrolled between December 7, 2022 and January 23, 2023 (16). Participants were divided into two groups by UDCA treatment status: UDCA-treated and none-UDCA-treated patients with chronic liver diseases. The none-UDCA-treated cohort with chronic liver diseases was 1:1 matched with the UDC-treated cohort based on age and gender. Each patient was followed up biweekly at Huashan Hospital throughout the study period.

Basic demographic and clinical information were obtained from Huashan Hospital. The clinical characteristics of SARS-CoV-2 infection were collected via outpatient clinics visits, telephone interviews and questionnaire in January 2023. SARS-CoV-2 infection was confirmed by positive results from either antigen or nucleic acid testing (17).

### Ethical considerations

This cohort study was approved by the Ethics Committee of Huashan Hospital (protocol number: KY2022-721).

### Inclusion and exclusion criteria

The inclusion criteria for patients with chronic liver diseases were as follows: (1) diagnosis of chronic liver diseases; (2) age ≥ 18 years old; (3) First SARS-CoV-2 infection between December 7, 2022 and January 23, 2023; (4) Willingness to participate the study. The exclusion criteria included: (1) uncertainty regarding prior SARS-CoV-2 infection status; (2) inability to respond to questions due to severe illness; (3) Refusal to participate in the study.

### Study variables and infection history

A comprehensive set of demographics, clinical, and laboratory variables was meticulously recorded for all enrolled participants. Demographic data included age (grouped as 18–40, 41–60, 61–80, and > 80 years), gender (female, *n*, %), and body mass index (BMI, kg/m^2^).

Additionally, vaccination status was classified into three groups: Not Fully Vaccinated, Fully Vaccinated, and Boosted. The number and type of vaccines received were recorded, including inactivated vaccines, mRNA vaccines, adenovirus vector vaccines, and subunit protein vaccines. The date of SARS-CoV-2 infection onset was documented, along with a detailed profile of clinical manifestations of the infection. Symptoms such as fever, fatigue, sore throat, cough, anosmia or ageusia, palpitations, musculoskeletal pain, and gastrointestinal symptoms (including diarrhea and vomiting) were recorded, along with their onset and duration.

The Not Fully Vaccinated group included individuals who either had not received any COVID-19 vaccination or had not completed the full vaccination regimen as defined by local health policies. The Fully Vaccinated group comprised individuals who had received two doses of a COVID-19 vaccine, with at least 14 days post-vaccination, or those who had completed the full vaccination course according to local guidelines. The Boosted group included individuals who had received a third or fourth dose of the COVID-19 vaccine, with at least 14 days since the most recent dose. Asymptomatic infection was defined as confirmed COVID-19 cases in which the individual exhibited no symptoms during the infection.

Disease diagnosis was recorded, including conditions such as Hepatitis B, Autoimmune Hepatitis, Primary Biliary Cholangitis, Fatty Liver, and other diseases. The fibrosis stage was classified according to the F0-F4 scale, based on liver biopsy or imaging results, with F0 indicating no fibrosis, and F4 representing cirrhosis. Fibrosis staging was further assessed using Masson’s trichrome–stained slides, with the METAVIR scoring system applied to determine the fibrosis stage (F0-F4) (23). In cases where the fibrosis stage was not be determined, the classification “Not clear” was used.

### Antiviral therapy

Data on antiviral therapy were collected to identify the types of antiviral agents prescribed to participants during the study period. This included various nucleos(t)ide analogs (NAs), such as Entecavir, Tenofovir disoproxil fumarate, Tenofovir alafenamide, and Tenofovir amibufenamide.

### Clinical indicators

Liver function was assessed including the following indicators: alanine aminotransferase (ALT), aspartate aminotransferase (AST), *γ*-glutamyl transpeptadase (GGT), alkaline phosphatase (ALP), total bilirubin, and albumin. Serum lipid parameters, including total cholesterol and triglycerides, were also recorded. Renal function was assessed using eGFR (ml/min/1.73 m^2^), while glucose levels were recorded as an indicator of metabolic function. The white blood cell count, hemoglobin, as well as platelet count were also collected to assess their blood routine status.

### Statistical analyses

All statistical analyses were conducted using R version 4.2.2 (R Foundation for Statistical Computing, Vienna, Austria) and RStudio (Posit, Boston, USA). Quantitative data are presented as means with standard deviations or medians with interquartile ranges, as appropriate. To compare enumeration data between groups with respect to component ratios and rates (percentages), the chi-square test or Fisher’s exact probability test were used. Data conforming to normal distribution and homogeneity of variance were compared using the independent cohort T test. For data with skewed distributions, the Mann–Whitney U test was used. A *p* value of less than 0.05 was considered statistically significant.

## Results

### Characteristics of patients with chronic liver diseases who were infected with SARS-CoV-2

**Figure 1:**
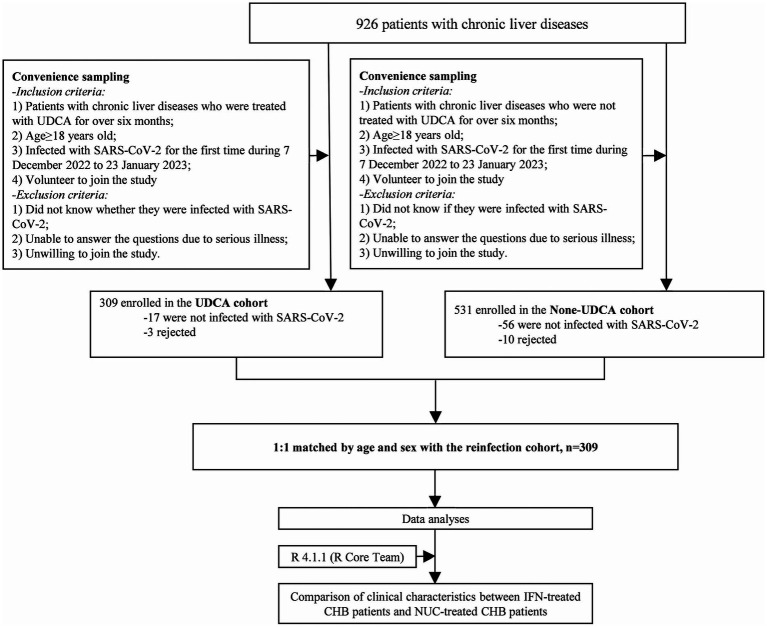
Flow charts of participants enrolment and study design.

A total of 926 patients with chronic liver diseases (including 458 patients with hepatitis B, 306 patients with autoimmune hepatitis, 38 patients with primary biliary cholangitis, 84 patients with fatty liver, and 40 patients with other diseases) who experienced their first SARS-CoV-2 infection during December 2022 to January 2023 were enrolled and divided into two groups based on UDCA administration: the UDCA-treated patients (*N* = 329) and the None-UDCA-treated ones (*N* = 531, Table 1; Figure 1). Subsequently, 309 patients from each group, matched 1:1 for age and gender, were enrolled as UDCA-treated and None-UDCA treated group, respectively (Figure 1). For matched groups, there were no statistical differences in vaccination status among two groups (Table 1). Interestingly, BMI presented higher levels in non-UDCA-treated group with less significant *p* value (*p* = 0.065).

**Table 1:** Characteristics of patients with chronic liver diseases and infected with SARS-CoV-2.

Characteristics	Cohort patients before enrollment			After propensity score matching		
	UDCA-treated (*n* = 329)	None-UDCA treated (*n* = 597)	*p*-Value	UDCA-treated (*n* = 309)	None-UDCA treated (*n* = 309)	*p*-Value
Age (years)	63 (55, 75)	56 (43, 59)	<0.001	62 (53, 77)	62 (44, 66)	0.978
18–40	28 (8.51)	25 (4.19)		25 (8.09)	25 (8.09)	
41–60	114 (34.65)	392 (65.66)		114 (36.89)	114 (36.89)	
61–80	153 (46.50)	136 (22.78)		136 (44.01)	136 (44.01)	
>80	34 (10.33)	44 (7.37)		34 (11.00)	34 (11.00)	
Female, *n* (%)	231 (70.21)	308 (51.59)	0.234	221 (71.52)	221 (71.52)	1
Body mass index (kg/m^2^)	23.5 ± 4.2	24.7 ± 4.5	0.057	23.8 ± 4.1	24.3 ± 4.2	0.065
Vaccination status			0.108			0.206
Not fully vaccinated	219 (65.57)	376 (62.98)		210 (67.96)	206 (66.67)	
Fully vaccinated	104 (31.61)	202 (33.84)		98 (31.72)	97 (31.39)	
Boosted	6 (1.82)	19 (3.18)		1 (0.32)	6 (1.94)	
Disease diagnosis type			<0.001			<0.001
Hepatitis B	85 (25.84)	373 (62.48)	<0.001	80 (25.89)	229 (74.11)	<0.001
Autoimmune Hepatitis	126 (38.30)	180 (30.15)	<0.001	121 (39.16)	36 (11.65)	<0.001
Primary Biliary Cholangitis	36 (10.94)	2 (0.34)	<0.001	36 (11.65)	2 (0.64)	<0.001
Fatty Liver	54 (16.41)	30 (5.02)	<0.001	46 (14.89)	30 (9.71)	<0.001
Other Diseases	28 (8.51)	12 (2.01)	<0.001	26 (8.41)	12 (3.88)	<0.001
Fibrosis stage			<0.001			<0.001
F0	90 (27.36)	202 (33.84)	<0.001	87 (28.16)	109 (35.28)	<0.001
F1	152 (46.20)	120 (20.10)	<0.001	145 (46.93)	67 (21.68)	<0.001
F2	35 (10.64)	79 (13.23)	<0.001	33 (10.68)	68 (22.01)	<0.001
F3	8 (2.43)	78 (13.07)	<0.001	3 (0.97)	32 (10.36)	<0.001
F4	5 (1.52)	45 (7.54)	<0.001	2 (0.64)	22 (7.12)	<0.001
Not clear	39 (11.85)	73 (12.23)	<0.001	39 (12.62)	11 (3.56)	<0.001
Antiviral therapy			<0.001			<0.001
Entecavir	21 (6.38)	89 (14.91)	<0.001	18 (5.83)	47 (15.21)	<0.001
Tenofovir disoproxil fumarate	7 (2.13)	65 (10.89)	<0.001	5 (1.62)	23 (7.44)	<0.001
Tenofovir alafenamide	99 (30.10)	278 (46.57)	<0.001	93 (30.10)	99 (32.04)	<0.001
Tenofovir amibufenamide	12 (3.65)	98 (16.42)	<0.001	8 (2.59)	87 (28.16)	<0.001
No antiviral therapy	190 (57.75)	67 (11.22)	<0.001	190 (61.49)	53 (17.15)	<0.001

All the data were collected between Dec 2022 and Jan 2023. Values are mean ± SD or number (percentage). SD, standardized difference; UDCA: Ursodeoxycholic acid. Not clear, the fibrosis grade was not assessed due to the absence of liver biopsy or Fibroscan data. Fibrosis stage; Masson’s trichrome–stained slides were evaluated to stage fibrosis using the METAVIR (F0-F4).

UDCA treatment was associated with a significantly higher rate of asymptomatic infections (30.10% vs. 6.47%, *p* < 0.0001) and lower levels of GGT and ALP (*p* < 0.001, Table 2). No significant differences were observed between the two groups in liver function markers such as ALT, AST, total bilirubin, and albumin, as well as renal function, glucose levels, and blood routine indices (Table 2). Triglycerides were notably higher in the UDCA group (*p* = 0.049, Table 2), while other lipid parameters presented no significant differences. Vaccination status and vaccine types did not differ between the groups.

**Table 2:** Comparison of the baseline laboratory variables and vaccination associated with asymptomatic SARS-CoV-2 infection among patients with chronic liver diseases.

Characteristics	UDCA-treated (*n* = 309)	Non-UDCA treated (*n* = 309)	*p*-value
Asymptomatic infections	93 (30.10)	20 (6.47)	<0.0001
Liver function indicators			
ALT (U/L)	25.03 (14.00, 32.00)	24.83 (16.00, 35.00)	0.879
AST (U/L)	22.11 (16.10, 35.00)	23.16 (21.01, 37.20)	0.786
GGT (U/L)	32.49 (16.00, 19.00)	42.49 (20.00, 119.05)	<0.001
ALP (U/L)	136.40 (77.03, 167.00)	96.40 (76.54, 161.21)	<0.001
Total bilirubin (mmol/L)	12.39 (10.30, 13.10)	15.92 (12.00, 26.45)	0.412
Albumin (g/L)	30.92 (37.40, 44.25)	34.92 (36.02, 48.17)	0.897
Serum lipid parameters			
Total cholesterol (mmol/L)	4.74 (4.39, 5.13)	4.62 (3.92, 5.21)	0.368
Triglyceride (mmol/L)	1.62 (1.29, 1.80)	1.27 (0.98, 1.66)	0.049
Renal function parameter			
eGFR (ml/min/1.73 m^2^)	99.38 ± 14.23	96.74 ± 13.37	0.256
Glucose indicator (mmol/L)	6.22 ± 1.02	6.12 ± 2.05	0.754
Blood routine index			
White blood cell (10^9^/L)	7.19 ± 2.10	6.95 ± 2.04	0.112
Hemoglobin (g/L)	135.60 ± 17.12	128.70 ± 17.12	0.573
Platelet (10^9^/L)	178.45 ± 84.33	170.32 ± 96.27	0.923
Vaccination status (*n*, %)			0.206
Not fully vaccinated	210 (67.96)	206 (66.67)	
Fully vaccinated	98 (31.72)	97 (31.39)	
Boosted	1 (0.32)	6 (1.94)	
Type of vaccine (*n*, %)			0.367
Inactivated vaccine	300 (97.09)	296 (95.79)	
mRNA vaccine	7 (2.27)	11 (3.56)	
Adenovirus vector vaccine	1 (0.32)	2 (0.65)	
Subunit protein vaccine	1 (0.32)	0	

All the data were collected between Dec 2022 and Jan 2023. Values are mean ± SD or number (percentage). SD, standardized difference; UDCA: Ursodeoxycholic acid.

### Risk factors for SARS-CoV-2 infection among patients with chronic liver diseases

**Figure 2:**
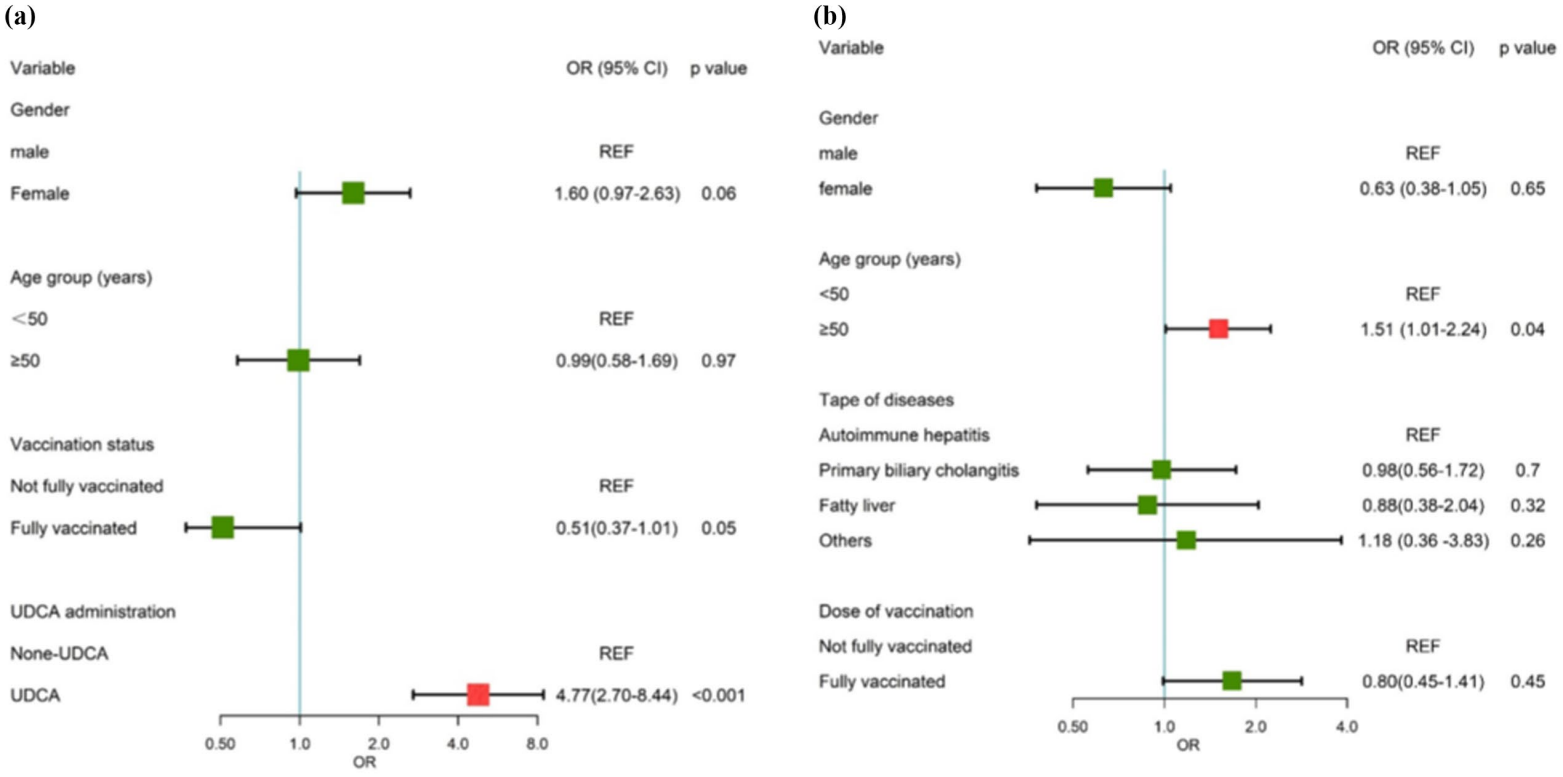
Forest map of risk factors. **(A)** The risk factor for asymptomatic SARS-CoV-2 infection among patients with chronic liver diseases. **(B)** The risk factors for the duration of the disease among ursodeoxycholic acid (UDCA)-treated patients with chronic liver diseases.

A total of 618 age and gender matched patients were included in the logistic regression analysis. Asymptomatic infection, gender, age, vaccination status, and type of treatment were included in the multivariable analysis. The results of the multivariate analysis revealed that UDCA administration and vaccination dose were protective factors against SARS-CoV-2 infection, with an OR of 4.77 (95% CI: 2.70–8.44, *p* < 0.001; 95% CI: 0.37–1.01, *p* = 0.05) (Table 3; Figure 2A).

**Table 3:** The multivariable logistic regression for assessing factors associated with asymptomatic SARS-CoV-2 infection among patients with chronic liver diseases.

Category	*p* value	OR (95%CI)
Gender		
Male		Ref (reference)
Female	0.06	1.60 (0.97–2.63)
Age group		
<50		Ref (reference)
≥50	0.97	0.99 (0.58–1.69)
Dose of vaccination		
Not fully vaccinated		Ref (reference)
Fully vaccinated	0.05	0.51 (0.37–1.01)
UDCA administration		
None-UDCA		Ref (reference)
UDCA	<0.001	4.77 (2.70–8.44)

UDCA, Ursodeoxycholic acid.

Age and gender had no significant association with asymptomatic infection among patients with chronic liver diseases, yet they were included in the final model to adjust for possible confounders.

### UDCA-treated patients with chronic liver diseases had less severe symptoms during SARS-CoV-2 infection

**Figure 3:**
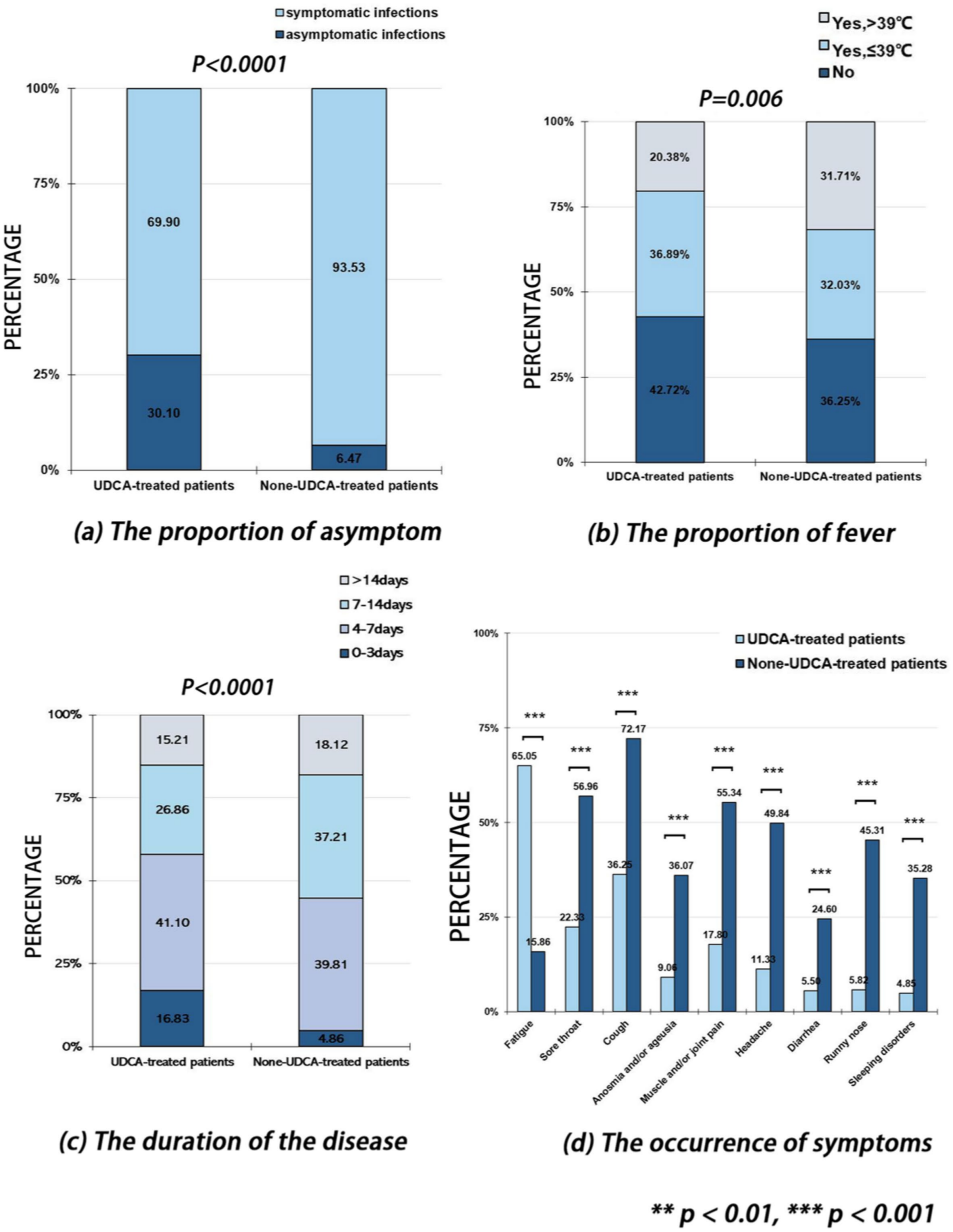
The proportion of asymptomatic SARS-CoV-2 infection, the proportion of fever, the duration of the disease, and symptom occurrence among patients treated with or without ursodeoxycholic acid (UDCA). **(a)** The proportion of asymptomatic SARS-CoV-2 infection; **(b)** The proportion of fever; **(c)** The duration of the disease; **(d)** The occurrence of symptoms.

The proportion of SARS-CoV-2 asymptomatic infection among UDCA-treated patients with chronic liver disease was 30.1%, which was significantly higher than None-UDCA-treated patients (6.47%) (*p* < 0.001, Supplementary Table S1; Figure 3A). UDCA-treated patients had a significantly milder severity of fever (*p* = 0.006) (Table 3; Figure 3B) and shorter duration of the disease (*p* = 0.001) (Table 3; Figure 3C) during SARS-CoV-2 infection, as well as reduced occurrence of sore throat (*p* = 0.001), cough (*p* < 0.001), anosmia and/or ageusia (*p* < 0.001), muscle and/or joint pain (*p* < 0.001), headache (*p* < 0.0001), runny nose (*p* < 0.001), and sleeping disorders (*p* < 0.001), compared to the None-UDCA-treated group (Supplementary Table S1; Figure 3D). Meanwhile, UDCA-treated patients also had fewer severe cases with pneumonia diagnosed (*p* = 0.026, Supplementary Table S1).

### Risk factors for the duration of disease among UDCA-treated patients with chronic liver diseases

Duration of the disease was included as an independent variable, while gender, age group, type of disease, vaccination status, and time of UDCA intake were considered as dependent variables in the multivariable analysis. Age greater than 50 years old is an independent risk factor for asymptomatic SARS-CoV-2 infection in UDCA-treated patients (OR = 1.51, 95% CI: 1.01–2.24, *p* = 0.04, Table 4; Figure 2B).

**Table 4:** The multivariable logistic regression for assessing factors associated with the duration of the disease among UDCA-treated patients.

Category	*P* value	OR (95%CI)
Gender		
Male		Ref (reference)
Female	0.70	1.22 (0.70–2.14)
Age group		
<50		Ref (reference)
≥50	0.04	1.51 (1.01–2.24)
Type of diseases		
Autoimmune hepatitis		Ref (reference)
Primary biliary cholangitis	0.70	0.98 (0.56–1.72)
Fatty liver	0.32	0.88 (0.38–2.04)
Others	0.26	1.18 (0.36–3.83)
Dose of vaccination		
Not fully vaccinated		Ref (reference)
Fully vaccinated	0.45	0.80 (0.45–1.41)

UDCA, Ursodeoxycholic acid.

Dose of vaccination, gender, and type of diseases had no significant correlation with asymptomatic infection among UDCA-treated patients, yet they were included in the final model to adjust for possible confounders.

## Discussion

In this study, we found that patients with chronic liver diseases were generally more susceptible to SARS-CoV-2 infection. Among the 926 patients with chronic liver diseases who were followed, 833 (89.96%) contracted COVID-19. Interestingly, UDCA was identified as a potential protective factor against SARS-CoV-2 infection, associated with a significant reduction in infection incidence among users, and notably higher rates of asymptomatic infections. In addition, UDCA-treated patients exhibited less severe symptoms during SARS-CoV-2 infection compared to the None-UDCA-treated group. Moreover, age over 50 years was identified as an independent risk factor for asymptomatic SARS-CoV-2 infection.

Although COVID-19 has been better managed and the death rate has significantly decreased, the highly transmissible SARS-CoV-2 variants continue to pose a global health threat. Effective management of COVID-19 and the reduction in mortality associated with SARS-CoV-2 infection have been largely achieved (24, 25). However, the emergence of the highly contagious SARS-CoV-2 variant has raised concerns and continues to threaten global public health (26, 27). To address the ongoing challenges, it is critical to explore and understand the role of vaccines in preventing and protecting against infection. Consistent with previous research, our findings highlight the importance of vaccines as the primary preventive measure for COVID-19. Vaccines have proven to be the main pillar of protection against infection (28, 29). Our study further underscores the vaccine’s role as an effective preventive measure against COVID-19 in patients with chronic liver diseases.

To note, our study found that UDCA was a protective factor against COVID-19 in patients with chronic liver diseases. The study suggested that the immunomodulatory properties of UDCA may play a role in reducing the risk of COVID-19. Previous studies have shown that UDCA can regulate immune responses (30–32), including reducing secretions of pro-inflammatory cytokines and enhancing function of regulatory T-cells. By modulating the immune response, UDCA may help mitigate the excessive immune response and cytokine storm observed in some cases of COVID-19 with severe syptoms. Secondly, the effect of UDCA on bile acid metabolism may influence susceptibility to SARS-CoV-2 infection. Bile acids have been shown to have antibacterial properties, including antiviral effects against other viruses (16–18). UDCA treatment may alter the composition and function of bile acids (33, 34), potentially affecting virus entry, replication, or host response to the virus.

In addition, the antiviral effect of UDCA may also result from its modulation of intracellular signaling pathways (35). Studies have shown that UDCA can inhibit the activation of nuclear factor κB (NF-κB) and mitogen-activated protein kinase (MAPK) pathways involved in inflammation and viral replication (35, 36). Moreover, ursodeoxycholic acid was reported to improve the cell migration of BEAS-2B human bronchial epithelial cells blocked by SARS-CoV-2 spike protein (37), which might also be closely related to the results of this study. By inhibiting these pathways, UDCA might impede viral replication. Therefore, our study showed that patients treated with UDCA experienced less severe symptoms of COVID-19, potentially by mitigating hepatic damage. This may be related to the role of UDCA in reducing disruptions in coagulative and fibrinolytic pathways, which are commonly observed in severe cases of the disease (38). Previous studies have shown that the pathogenesis of COVID-19 involves two key processes: viral replication in the early phase and a dysregulated immune/inflammatory response leading to systemic tissue damage in the later phase (39, 40). Our findings suggest that UDCA may play a potential role in modulating immune and inflammatory responses, helping to mitigate disease severity in the later stages.

The study primarily investigated the therapeutic role of UDCA in alleviating the clinical severity of COVID-19 in patients with chronic liver diseases. Previous research has suggested that UDCA may reduce SARS-CoV-2 entry via ACE2 downregulation and mitigate liver damage associated with systemic inflammation during infection (41). It is previously demonstrated that other hepatoprotective drugs, such as obeticholic acid (OCA) (42) and glycyrrhizic acid (GA) (43), also presented anti-inflammatory, antifibrotic, and direct antiviral effects in COVID-19 patients. Given the interplay between liver disease severity and COVID-19 outcomes, fibrosis staging remains a crucial factor, as advanced fibrosis is known to intensify systemic inflammation and worsen clinical prognosis in viral infections, including COVID-19 (44, 45). Although our study did not yield statistically significant results based on fibrosis staging, this factor remains a critical consideration for future research aimed at stratifying patients and enhancing our understanding of their responses to hepatoprotective and antiviral therapies. While these findings highlight potential therapeutic avenues, there is a notable paucity of comparative clinical studies evaluating the efficacy of these approaches in COVID-19 patients. Future investigations should focus on clarifying the role of these therapies in mitigating hepatic injury and inflammation, particularly through large-scale, multi-center trials. Such studies have the potential to inform the development of more effective adjunctive strategies for managing COVID-19 in patients with pre-existing liver conditions.

Our study explored the clinical effects of UDCA on COVID-19 infection. However, there are some limitations. First, this is a single-center study which might be variety deficient. Second, the size of the cohort was limited, which might lead to potential bias. Additionally, the severity of liver disease was not systematically assessed, which could have offered more insights into the variability of patient responses and outcomes. Future studies should take these factors into account to better understand their impact on treatment efficacy. Moreover, larger multi-center studies with more diverse patient populations are needed to validate and extend our findings.

In conclusion, our study provided the evidence that UDCA was a potential protective factor against COVID-19 in patients with chronic liver diseases. This finding highlighted the potential benefits of UDCA therapy in the management of patients infected with COVID-19 in order to relieve the severity of the symptoms. Further studies would be carried out to clarify the underlying processes and assess the therapeutic potential of UDCA in administration of COVID-19.

## Conclusion

The study demonstrated the clinical characteristics of SARS-CoV-2 infection in patients with chronic liver diseases, and found that UDCA was a protective factor which can relieve severity of symptoms during COVID-19. Due to an indestructible persistent mutation of SARS-CoV-2, monitoring the occurrence and severity of infection is critical. For patients with chronic liver diseases complicated with SARS-CoV-2 infection, more attention should be paid to the clinical management of patients. In the future, more clinical studies are needed to determine whether UDCA is an effective treatment of COVID-19 in patients with chronic liver diseases.

## Data Availability

Publicly available datasets were analyzed in this study. This data can be found at: https://yxky.fudan.edu.cn.
