# Supplementary material for: Ursodeoxycholic acid relieves clinical severity of COVID-19 in patients with chronic liver diseases
Source: Front Med (Lausanne). 2025 Feb 6;12:1494248. doi: 10.3389/fmed.2025.1494248 (PMC11839632; doi:10.3389/fmed.2025.1494248)
Supplement: Supplementary file 1 [file Table_1.docx]

| Clinical manifestation | UDCA-treated patients  (N=309) | None-UDCA-treated patients  (N=309) | *P* value |
| --- | --- | --- | --- |
| Asymptomatic infections | 93 (30.10) | 20 (6.47) | <0.0001 |
| With pneumonia |  |  | 0.026 |
| Severe | 1(0.3) | 9 (2.9) |  |
| Non-severe | 308 (99.7) | 300 (97.1) |  |
| Duration of disease (days) |  |  | <0.0001 |
| 0-3 | 52 (16.83) | 15 (4.85) |  |
| 4-7 | 127 (41.10) | 123 (39.81) |  |
| 7-14 | 83 (26.86) | 115 (37.21) |  |
| >14 | 47 (15.21) | 56 (18.12) |  |
| Fever |  |  | 0.006 |
| No | 132 (42.72) | 112 (36.25) |  |
| Yes, ≤39℃ | 114 (36.89) | 99 (32.03) |  |
| Yes, >39℃ | 63 (20.38) | 98 (31.71) |  |
| Fatigue | 201 (65.05) | 49 (15.86) | <0.0001 |
| Sore throat | 69 (22.33) | 176 (56.96) | <0.0001 |
| Cough | 112 (36.25) | 223 (72.17) | <0.0001 |
| Anosmia and/or ageusia | 28 (9.06) | 96 (36.07) | <0.0001 |
| Muscle and/or joint pain | 55 (17.80) | 171 (55.34) | <0.0001 |
| Headache | 35 (11.33) | 154 (49.84) | <0.0001 |
| Diarrhea | 17 (5.50) | 76 (24.60) | <0.0001 |
| Runny nose | 18 (5.82) | 140 (45.31) | <0.0001 |
| Sleeping disorders | 15 (4.85) | 109 (35.28) | <0.0001 |

**Supplementary Table 1:** Comparative Clinical Characteristics of UDCA-treated and Non-UDCA-Treated patients with chronic liver diseases co-infected with SARS-CoV-2.

*All the data were collected between Dec 2022 and Jan 2023.*

*Values are mean ± SD or number (percentage).*

*SD, standardized difference; UDCA: Ursodeoxycholic acid.*
